# Latency and interval therapy affect the evolution in metastatic colorectal cancer

**DOI:** 10.1038/s41598-020-57476-y

**Published:** 2020-01-17

**Authors:** Hamid Nikbakht, Selin Jessa, Mahadeo A. Sukhai, Madeleine Arseneault, Tong Zhang, Louis Letourneau, Mariam Thomas, Mathieu Bourgey, Michael H. A. Roehrl, Robert Eveleigh, Eric X. Chen, Monika Krzyzanowska, Malcolm J. Moore, Amanda Giesler, Celeste Yu, Philippe L. Bedard, Suzanne Kamel-Reid, Jacek Majewski, Lillian L. Siu, Yasser Riazalhosseini, Donna M. Graham

**Affiliations:** 1 Department of Human Genetics, McGill University, Montreal, Québec Canada; 2 McGill University and Génome Québec Innovation Centre, Montreal, Québec Canada; 3 Princess Margaret Cancer Centre, Toronto, Ontario Canada; 4 UHN Program in BioSpecimen Sciences, Toronto General Hospital, Toronto, Ontario Canada; 5 Department of Pathology, Toronto General Hospital, Toronto, Ontario Canada

**Keywords:** Cancer genomics, Molecular medicine

## Abstract

While comparison of primary tumor and metastases has highlighted genomic heterogeneity in colorectal cancer (CRC), previous studies have focused on a single metastatic site or limited genomic testing. Combining data from whole exome and ultra-deep targeted sequencing, we explored possible evolutionary trajectories beyond the status of these mutations, particularly among patient-matched metastatic tumors. Our findings confirm the persistence of known clinically-relevant mutations (e.g., those of RAS family of oncogenes) in CRC primary and metastases, yet reveal that latency and interval systemic therapy affect the course of evolutionary events within metastatic lesions. Specifically, our analysis of patient-matched primary and multiple metastatic lesions, developed over time, showed a similar genetic composition for liver metastatic tumors, which were 21-months apart. This genetic makeup was different from those identified in lung metastases developed before manifestation of the second liver metastasis. These results underscore the role of latency in the evolutionary path of metastatic CRC and may have implications for future treatment options.

## Introduction

Results of somatic mutational analysis increasingly guide treatment decisions in oncology. In colorectal cancer (CRC), activating mutations in *KRAS* (codons 12, 13, 61, 117 and 146), *NRAS* (codons 12, 13, 61, 117 and 146), and *BRAF* (V600E) confer resistance to anti-epidermal growth factor receptor (EGFR) therapy including Cetuximab and Panitumumab^1–4^. This has resulted in extended *RAS* testing to more comprehensively determine treatment sensitivity in CRC^5,6^. Additionally, microsatellite instability (MSI) caused by deficiency of mismatch repair (MMR) proteins, has predictive value for response to adjuvant chemotherapy and immune checkpoint inhibitors in CRC^7,8^. Comparison of primary and metastatic tumors within individual patients underscores the heterogeneous nature of tumors and the varying responses of sub-clones to different treatment strategies^9^. Despite increasing use of molecular testing into clinical practice, this is typically performed using tumor tissue from a single anatomic location, at a single time point, obtained from archived diagnostic specimens or a freshly procured biopsy. Genomic evaluations of tumor tissue specimens from different sites within tumor or over time are rarely performed, due to several factors including risks associated with invasive procedures, cost, sample accessibility, and DNA quality. Current analyses of primary and metastatic CRC indicate low rates of variation between tumor sites for the clinically-relevant mutations that may impact upon treatment decisions^10,11^. However, these comparisons have surveyed the presence or absence of somatic mutations without specifically assessing their allelic frequency, which can reflect on genomic evolution of metastatic CRC. Likewise, our knowledge about the extent to which systemic therapy can influence this evolutionary path is limited. Here, we explore the evolution of metastatic CRC by analyzing the dynamic changes in frequency of somatic mutations identified through whole exome sequencing (WES) of patient-matched primary and metastatic tumors. Investigating the genetic architecture of different metastatic lesions from the same patient in the presence or absence of systemic therapy provides insight into both inherent and therapy-driven evolutionary paths. We further study the contribution of specific mutations in six clinically-relevant genes (*APC, TP53, KRAS, NRAS, PIK3CA* and *BRAF*) to the mutational burden of CRC by measuring variant allele frequencies using targeted ultra-deep sequencing.

## Methods

### Patients and samples

The study cohort comprised 350 patients with advanced malignancies enrolled in the initial phase of the Integrated Molecular Profiling in Advanced Cancer Trial (IMPACT) at the Princess Margaret Cancer Centre (NCT 01505400)^12^. All patient information and tissue were collected under the University of Toronto/University Health Network Research Ethics review board-approved protocol (REB#12-5453-TE) with written informed consent obtained from all participants. We reviewed medical records of these patients and selected those with multiple tumor samples (n = 62) and with CRC as the primary diagnosis (n = 27). From these, 15 patients had tissue available from multiple tumor samples with 11 having more than one tumor sample with adequate tissue for evaluation (26 samples in total) and were included in the current study (Supplementary Fig. 1). The cohort of 11 patients included eight patients with available tissue from the primary tumor and ≥1 metastatic site, two patients with pairs of metastases only, and one patient with an anastomotic recurrence five years after initial resection of the primary tumor (Supplementary Table 1). One sample from each patient had previously been genotyped using a customized 23-gene panel via the Sequenom MassARRAY^12^. All procedures and experiments were conducted in accordance with University of Toronto/University Health Network guidelines.

### Tissue preparation and targeted capture

Representative slides or tissue blocks from tumor specimens were collected. A hematoxylin and eosin–stained section from each tumor was reviewed by a specialized gastrointestinal pathologist. Tumors were macrodissected to maximize tumor content where necessary, and the percentage of viable tumor cells was estimated by the pathologist. DNA was isolated from the 36 available formalin-fixed paraffin embedded (FFPE) tissue specimens using QIAamp DNA micro kit (QIAGEN Inc, Valencia, CA), following instructions provided by the manufacturer. Loci harboring clinically-relevant mutations of *TP53* (codons 175, 213, 245, 248, 273 and 306), *APC* (codons 1378 and 1450), *KRAS* (codons 12, 13, 22, 61, 117 and 146), *NRAS* (codons 12, 13 and 61), *PIK3CA* (codons 539, 542, 545 and 1047), and *BRAF* (codon 600) were captured by multiplex amplification on Fluidigm access arrays (South San Francisco, CA) using 50 ng of DNA. PCR primers specific to each locus are provided in Supplementary Table 2. Hotspots were selected following literature review and use of publicly available datasets, with input from our molecular diagnostics laboratory^3,4,13–15^. Of note, *NRAS* codon 117 and 146 mutations were not included in this analysis due to their relative rarity^4^.

### Whole-exome sequencing and data analysis

WES libraries were generated using the Nextera Rapid Capture Enrichment library preparation kit (Illumina) according to the manufacturer’s recommendations and were sequenced on an HiSeq. 2500 paired-end 100 base pair reads. Quality controlled reads processed by trimmomatic^16^ (v0.32) to remove adapters and low quality reads were aligned to the human genome build GRCh37 using bwa-mem^17^ (version 7.6). Mapped reads were further refined using GATK^18^ (v3.2.2) and Picard program suites^19^ (v1.118) to improve mapped reads near indels (GATK indel realignment) and improve quality scores (GATK base recalibration) as well as mark duplicate reads with the same paired start locations (Picard mark duplicates). Somatic calls generated using Mutect (v1.1.6) for SNVs and scalpel (v0.5.2) for indels^20^ were further processed with the addition of functional annotations using snpEff ^21^ (v3.6b) and genomic annotation using Gemini^22^ (v0.11.1a). All non-silent variants were inspected manually for quality control using Integrative Genome Viewers (IGV)^23^, and were predicted for functionality using CADD^24^, and fitness consequence scores^25^.

### Mutational signature analysis

Mutational signature analysis was performed individually for each lesion using somatic base substitutions identified in that lesion by Mutect. The R packages SomaticSignature and BSgenome.Hsapiens.1000genomes.hs37d5^26^ were used to extract the somatic mutational profiles and to provide the frequency for each of the 96 possible single base substitutions and their context combinations. Individual mutational profiles were then compared against the known signatures described by Alexandrov *et al*. using the DeconstructSigs R package to infer the contribution of known reference signatures to the individual somatic mutation profiles^27,28^.

### Copy number aberration analysis

To analyze copy number variations in our samples we used an in-house program that exploits deviation of B allele frequency from 50% as well as read-depth, normalized based on the average coverage using WES data. Copy number variation (CNV) events were defined as follows: deviation from 50% in B allele frequency and a significant increase in normalized coverage was considered as a sign of amplification, deviation from 50% in B allele frequency, however, if was accompanied by decrease in normalized coverage, would be considered as deletion. If there was no change in the normalized coverage while B allele frequency represents deviation from 50%, we called it a potential copy-neutral loss of heterozygosity (LOH).

### Inferring clonal evolutionary trajectories within patients

We used allele frequency values corrected^29^ for both the tumor content and CNV events as the raw input to infer sub-clonal composition and evolutionary trajectories within each patient. We corrected the original allele frequency values based on the copy number aberration events harboring these variations. In our correction, we use the naïve assumption that the copy number aberration always affects the frequency of the altered allele to the largest extent. For example, in case of duplication, we assume the duplication occurred on the chromosome harboring the altered allele. Thus, we divide the number of altered allele reads into half to correct for the duplication and calculate the allele frequency (Duplication: Ref’ = Ref; Alt’ = Alt/2). In the case of deletion, we assume the deletion happens on the chromosome with reference allele. Thus, to correct for this, we add the number of reference allele reads to the number of altered allele reads and count it as the final number of reference allele read count (Deletion: Ref’ = Ref + Alt; Alt’ = Alt). For a gene on chromosome X (in male patients), this is treated as a loss of heterozygosity case. In this case we add the number of alternate reads to the twice number of reference reads and use it as new reference read counts (Gene on X in male patients: Ref’ = Ref *2 + Alt; Alt’ = Alt). In cases of copy neutral loss of heterozygosity, we add half of alternate reads to the number of reference reads and use it as the new value for reference read counts (Copy Neutral LOH: Ref’ = Ref + Alt/2; Alt’ = Alt/2). We developed an in-house program to cluster recurrent and exclusive mutations in primary versus metastatic lesions using expectation maximization algorithm using EMClust^30,31^ library in R^32^ and used the resulting putative subpopulations, their relative frequencies, and information about therapy administered between resections to infer evolutionary trajectories between lesions. The funnel plots representing these proposed scenarios are drawn manually using Adobe Photoshop^©^ (CS3 extended version) program. The clustering plots representing subpopulations inferred from somatic mutations are created using ggplot2^33^ library.

### Accession codes

Raw sequence data have been deposited in the European Genome-phenome. Archive (EGA), under the Accession Code EGAS00001003646.

## Results

### Patient samples and whole exome sequencing

We analyzed primary CRC, matched blood, and metastatic tumors (where available) from 11 patients, from which, all had germline DNA from blood samples, seven from both primary and more than one metastatic tumors, one from only the primary tumor, one from a primary tumor and an anastomotic recurrence, and two from metastatic tumor pairs only. Clinical characteristics and patient treatment strategies are outlined in Supplementary Table 1. Targeted ultra-deep sequencing (average of 25000×) was applied to clinically-actionable mutations for validation and measuring mutant allele frequencies. WES (see Methods) was applied to DNA isolated from macrodissected formalin-fixed paraffin-embedded (FFPE) tumor specimens and blood samples to an average coverage depth of 63X and 95X for blood and tumor samples, respectively.

### Somatic mutations

On average, we detected 116 and 156 somatic exonic mutations in primary and metastatic lesions, respectively (excluding the hypermutated primary tumor in REACT-009-A, which harbored 1285 somatic mutations) (Supplementary Table 1).

We investigated global mutational patterns by analyzing base-substitution profiles and mutational signatures in tumor samples^27,34^ (Supplementary Table 3). As reported recently, mutational signatures can shed light on mutagenic processes that affect cancer genomes. We observed that the primary tumor in patient-009 (REACT-009-A) and metastatic samples REACT-010-B and E are enriched for signature 6 (Supplementary Fig. 2). This signature is characterized by Cytosine deamination in repeated sequences with NpCpG pattern, and is associated with the inactivation of DNA mismatch repair (MMR) genes in colorectal cancer. Deficiency of MMR proteins results in microsatellite instability (MSI) pattern^27^ which is reported in a variety of cancers including CRC, and with prognostic and predictive implications for CRC^8,34,35^. Our WES results from patient-009’s primary tumor confirmed a hypermutated genome in this tumor, with mutations in the MMR genes *MSH2* and *MSH5* (Supplementary Tables 1 and 4).

**Figure 1:**
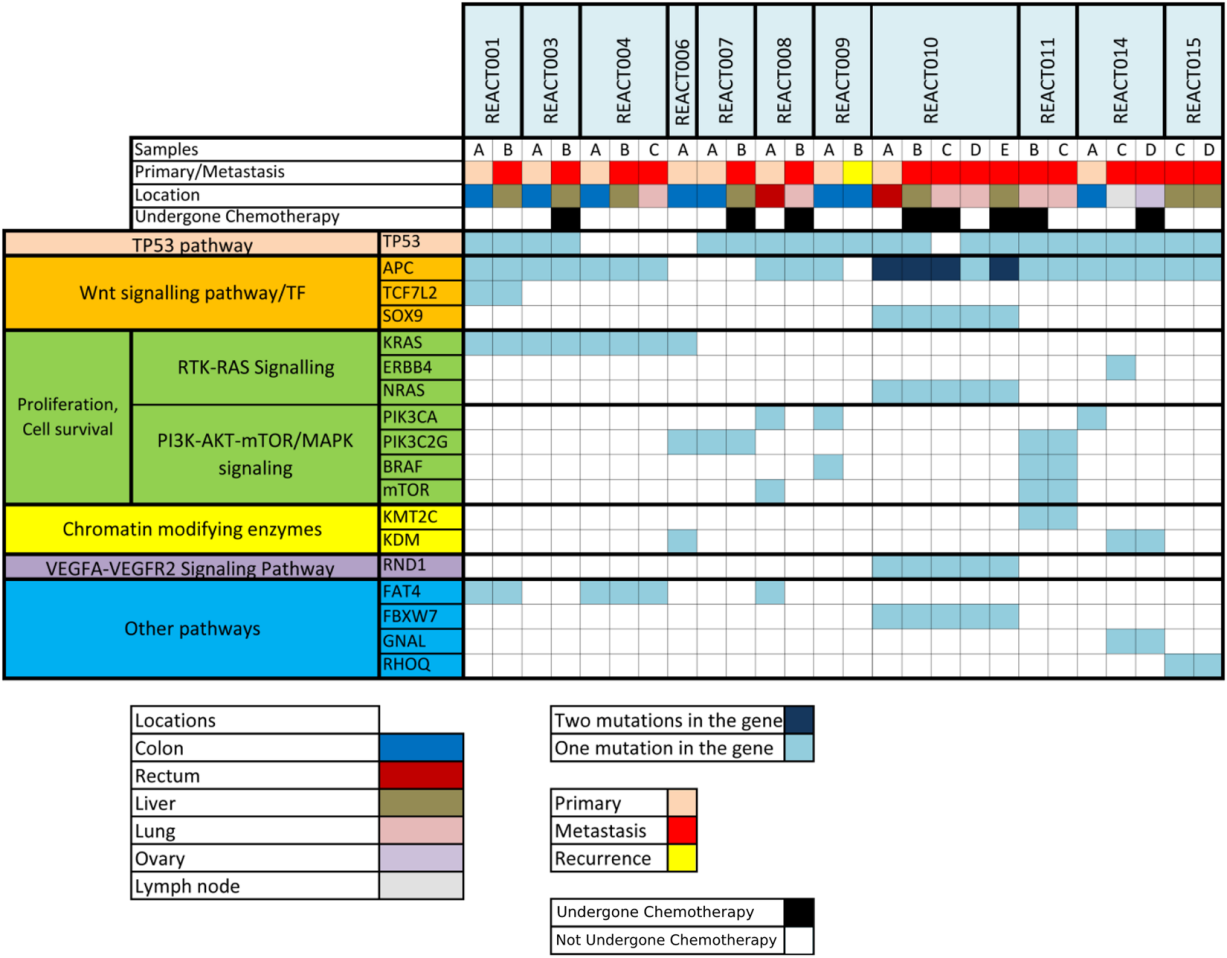
Mutational status of genes involved in CRC pathways across samples studied. Patient IDs are shown on top and individual samples procured from each patient are depicted beneath the corresponding patient ID. The type of sample (primary or metastasis), affected organ and information about the application of chemotherapy prior to the sampling are provided in colored legends.

Next, we focused on non-silent somatic mutations in primary tumors, and identified 1116 such mutations affecting 1019 genes following manual *in silico* verification of mutations using Integrative Genomics Viewer (IGV)^23^ (Supplementary Table 4). Forty-five of the affected genes were recurrently mutated in at least two primary tumor samples (Supplementary Table 4), among which, nine genes were affected in the primary tumor of at least 3 of the 9 patients whose primary tumors were analyzed by WES (33.3%); *APC, TP53, KRAS, SYNE1, FAT4, FBN2, PIK3CA, UHRF1* and *WDFY4*. Among these 9 patients, *APC* and *TP53* were the most frequently mutated genes affected by somatic mutations in 78% followed by *KRAS* which was mutated in 44% of primary tumors (Fig. 1), in line with previous studies reporting these genes as the most commonly mutated genes in CRC^36,37^.

All *APC*-mutated tumors were affected with at least one protein truncating mutation, which may lead to the activation of WNT pathway^38^. *TP53* was affected in nine patients in our study, with various mutations. Activating mutations of *KRAS* codon 12 were detected in four patients, and oncogenic mutations of *NRAS* (G13V) and *BRAF* (V600E) were each identified in one additional tumor, contributing to a prevalence of 54.4% for activating mutations of RAS pathway. Notably, these genes were not mutated concurrently in tumors highlighting a mutually-exclusive pattern for RAS pathway mutations (Fig. 1 and Supplementary Table 4). Three patients were affected with *PIK3CA* mutations, two of which (E545G and Q546K) have been previously reported in CRC^39^. A third *PIK3CA* mutation (H665D) with uncertain clinical significance has no previous record in COSMIC database, but was predicted as likely functional based on CADD and fitCons scores. Three additional tumors were affected with mutations in *PIK3C2G*, resulting in a frequency of 55.5% of primary samples affected with PI3K pathway mutations (Fig. 1 and Supplementary Table 4).

Among other recurrently mutated genes, *SYNE1* and *FAT4* have been reported with high prevalence of non-silent mutations in CRC patients^36^. Hypermethylated-SYNE1 promoter has been found in 80% of patients with colitis-associated CRC^40^ Likewise, UHRF1 has been shown to be involved in cellular proliferation and molecular pathogenesis of CRC in the right hemicolon^41^.

### Chromosomal copy number alterations

Analysis of the chromosomal copy number alterations (CNAs) revealed highly recurrent amplifications of 13q in 75% (6/8) of primary tumors. This was followed by gains of 11q and 6q in 50% (4/8) and 37.5% (3/8) of primary tumors, respectively. Moreover, we detected 63% (5/8) cases of LOH and one case of deletion of 17p in a primary tumor (Supplementary Table 5).

Amongst seven patients with both primary and metastatic tumors, 3 patients showed the same CNA in both primary tumor and one or more metastatic lesion(s). These include recurrent amplifications of 6q and 13q (REACT-001 and REACT-010) as well as copy neutral loss of heterozygosity in 17p (REACT-004 and REACT-010) (Supplementary Table 5). Patient REACT-011 shows a very similar CNA pattern between its two metastases (AMP at 3p, 5p, 6p, 6q, 11q, 12q, 13q, 19p and 21q.) This CNA similarity pattern between metastases was less pronounced in other patients. This patient also had similarity in non-silent somatic mutation patterns between the metastatic samples. There was a short time interval (6-months) between metastatic lesions for patient REACT-011 during which no treatment was administered. Our results show that patients who had received an interval treatment seem to show a greater decrease in the total number of complete arm aberrations, that is loss or gain of a complete chromosome arm, between their samples (REACT-007, REACT-008 and REACT-014) compared to patients who did not receive any treatment (REACT-011 and REACT-015) (Supplementary Table 5). Effects of therapy on the abundance of CNVs in post-treatment liver metastases have previously been reported^42^.

### Analysis of the clonal patterns

We investigated the clonality of tumor samples and inferred evolutionary trajectories of metastatic tumors in patients with paired primary and metastatic lesions using allele frequency of somatic mutations (see Methods). Our analysis confirmed sub-clonal composition of both primary and metastatic tumors across all samples. Overall, whereas private mutations (i.e., mutations exclusive to primary or metastatic lesion) constituted a large proportion of somatic mutations in each specimen, mutations in known driver genes were consistently present in both primary and metastatic lesions, with few exceptions discussed below.

### Patients who received no interval treatment

**Figure 2:**
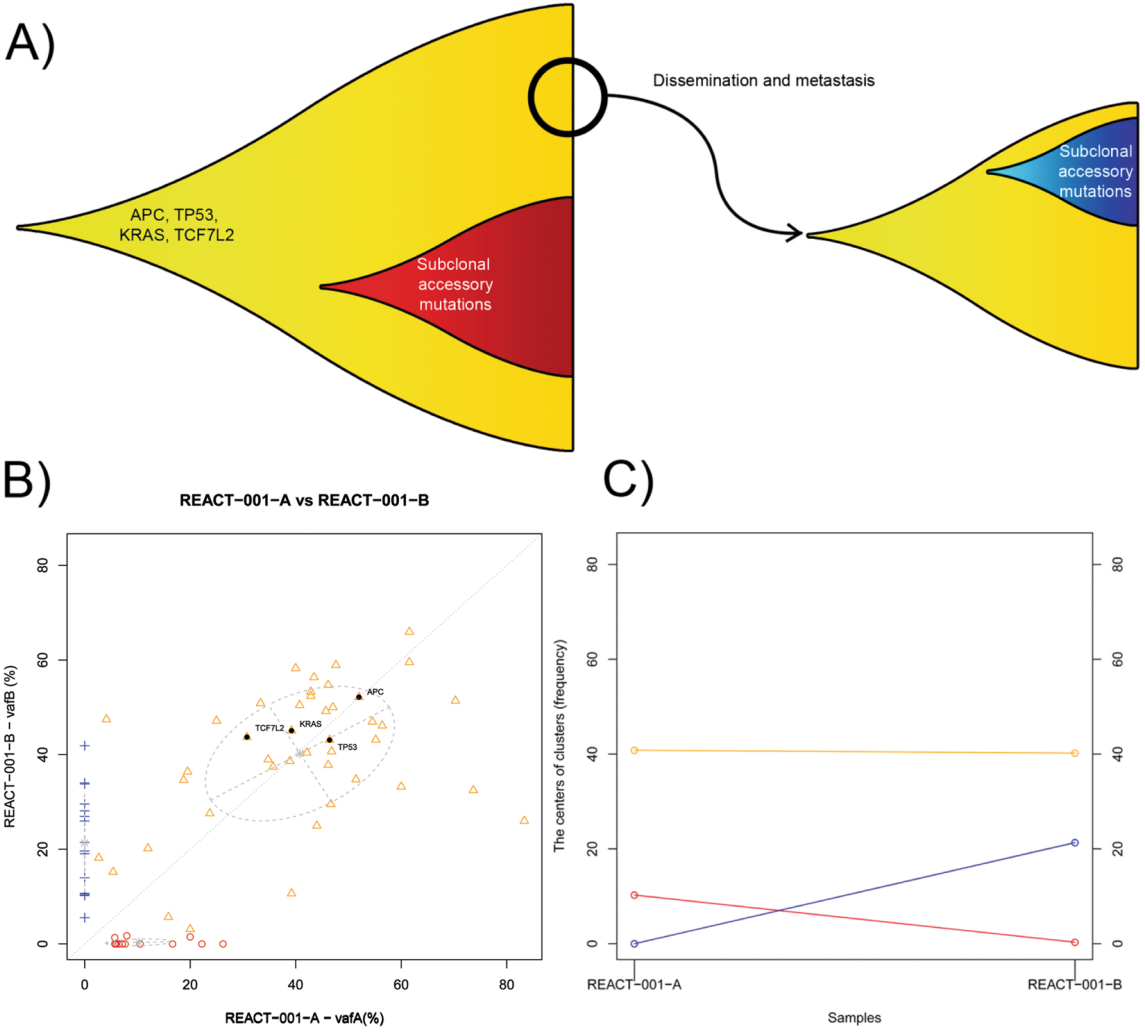
(**A**) Proposed scenario for the evolution and clonality within the primary and metastatic lesions in patient REACT-001. B and C represent the clustering of somatic mutations in these two tumor samples, and the changes in allele frequency of the centers of these clusters. The center of a cluster inferred from somatic mutation allele frequencies corresponds to the average cellular proportion of the subpopulation characterized by mutations in that cluster. The allele frequency values were inferred from WES data, and corrected for CNA events and for tumor content, which was estimated from the histological examinations. (**B**) Clustering of the mutations in primary versus metastatic lesion into groups which characterize clonal subpopulations in the lesions. (**C**) Changes in the population proportion in different clusters. The yellow cluster maintains a ~ 40% cell population proportion while the red cluster of mutations is lost in the metastatic lesion and the new cluster (sub-clone) of mutations has arisen in the metastatic lesion (blue). Red subpopulation (exclusive to the primary tumor), harbor mutations accumulated in a subpopulation different from which the dissemination of metastatic cells happens or potentially emerged after the dissemination. The blue cluster (exclusive to the metastatic lesion), however, represent cells with new mutations emerging following dissemination from the primary tumor.

Analysis of primary tumor and metastases in patients who had received no interval therapy revealed consistency for presence and relative frequency of identified driver mutations. For example, in patient REACT-001, where no interval chemotherapy was administered, the relative frequency of main driver mutations (*APC* E1288*, *TP53* L125LH, and *KRAS* G12A) did not change between the primary and metastatic tumors, suggesting that the cluster of cells disseminated from the primary tumor to form the metastatic tumor harbored the entire set of main driver mutations (i.e. the dissemination happens after the occurrence of all above mentioned alterations) (Fig. 2A). In both primary and metastatic tumor samples, some sub-clonal (passenger) mutations^29^ emerged secondary to the main driver mutations. Similar evolutionary trajectories are presented in Supplementary Fig. 3 for patient REACT-004 who had not received interval therapy. The lack of multi-region sequencing in each of these samples leaves sampling bias as a potential alternative explanation.

### Patients who received interval treatment

**Figure 3:**
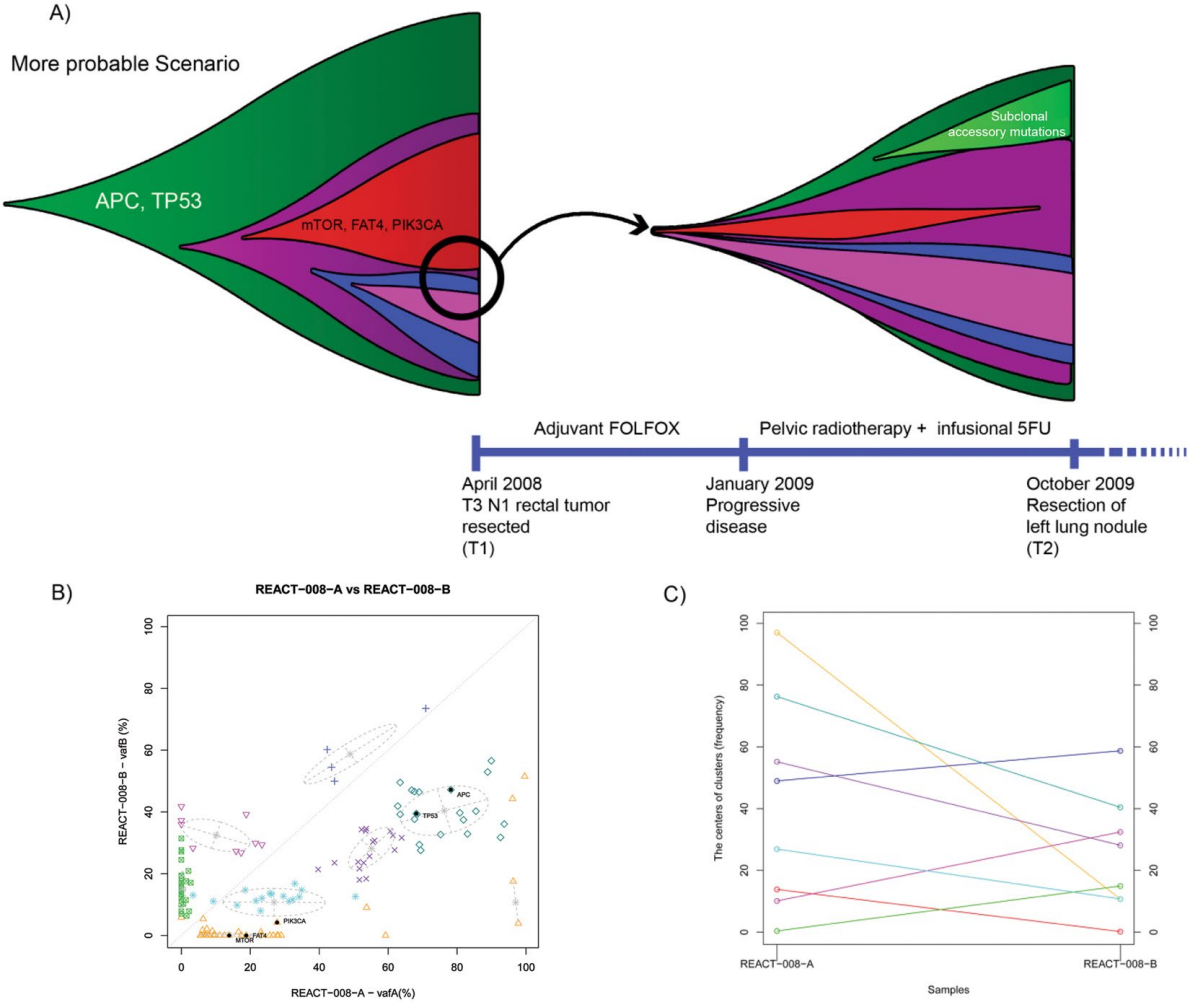
(**A**) Proposed scenario for the evolution and clonality within the primary and metastatic lesion in patient REACT-008. (**B**) Clustering of the mutations in primary versus metastatic lesions. (**C**) Changes in the population proportion in different clusters.

A different pattern is evident following the analysis of cases where interval systemic treatment has been administered. Patient REACT-008 was diagnosed with a primary tumor in the rectum, and had treatment with adjuvant folinic acid, fluorouracil, and oxaliplatin (FOLFOX) between resection of the primary and a lung metastasis. In this case, the primary tumor had been completely resected with no residual disease detected at the time of chemotherapy administration. *TP53* (R114H) and *APC* (E1379*) mutations exist in a sub-clone present in relatively high frequency in both primary and metastasis. However, relative abundance of a fraction of cells harboring *PIK3CA* (H665D) and *mTOR* (V1795M) mutations, which are predicted as likely functional mutations, declined substantially in the lung metastasis compared to the primary tumor (Fig. 3). This could be due to either cytotoxicity on the metastatic tumor or the absence of these mutations in the cluster of cells that disseminated from the primary tumor to form the lung metastasis. Interestingly, the most frequent cell population shows a relatively stable frequency in the metastatic lesion, which may be explained by the presence of the *TP53* mutation, possibly conferring resistance to chemotherapy in these cells. The possible elimination of the sub-clone harboring mutations in *mTOR*, *PIK3CA* and a *FAT4* due to adjuvant chemotherapy could have happened either in the primary tumor or in the metastasis. Given the chronology of systemic therapy in this case, the latter scenario seems more likely than the former. This scenario is represented in the funnel plot (Fig. 3).

**Figure 4:**
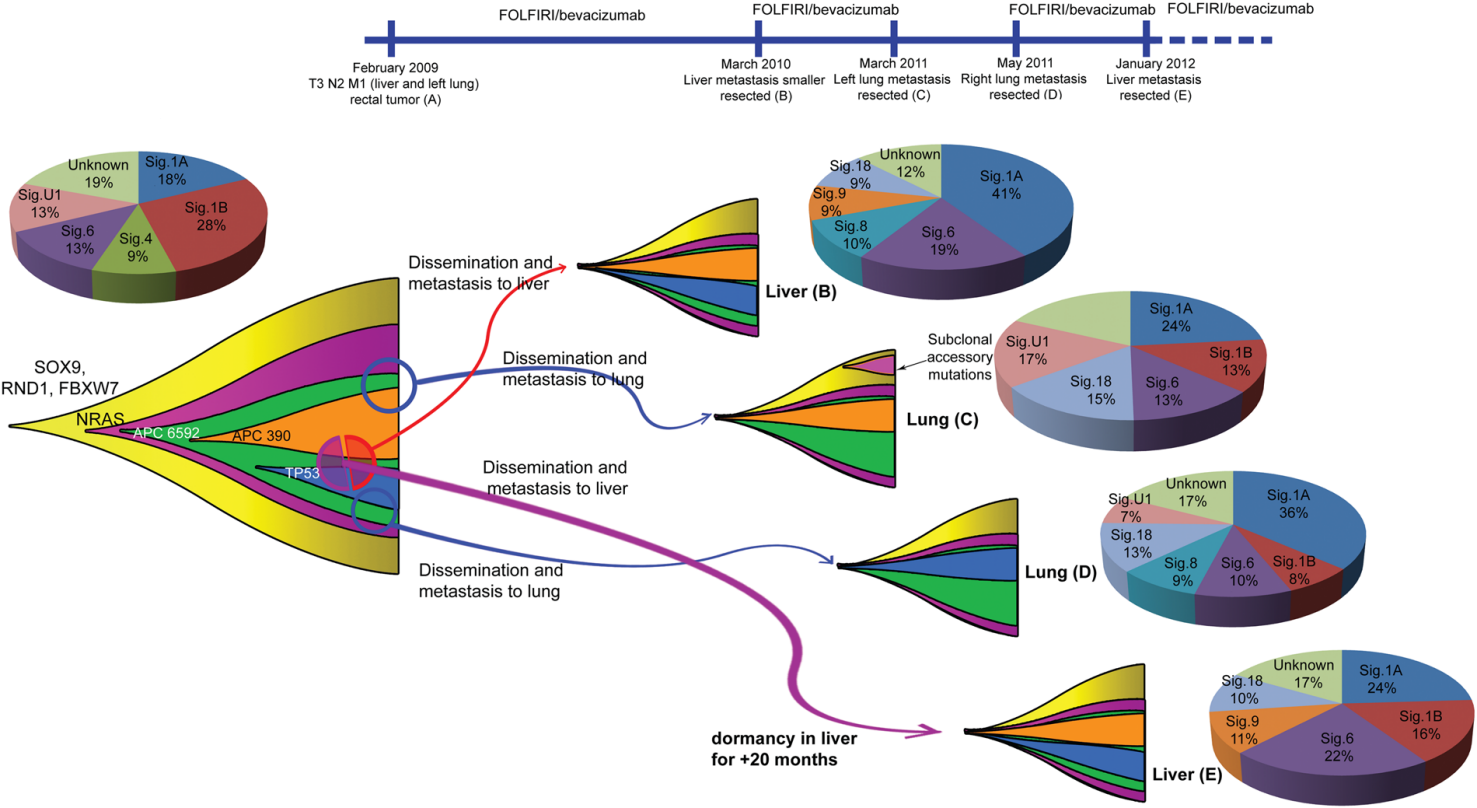
Proposed evolution and clonality and changes in the mutation signature within primary tumor and four metastatic lesions in patient REACT-010.

Patient REACT-010 is interesting due to the number of metastatic samples and the timing of systemic therapy administered. This patient has a primary tumor in the rectum, and two pairs of metastasis, one pair in the liver (21-months apart) and one pair in the lungs (left and right; one-month apart) (Fig. 4). The patient received folinic acid, fluorouracil, and irinotecan (FOLFIRI)/bevacizumab treatment between the primary and the first metastasis (liver REACT-010-B), and between the first and second metastasis (left lung REACT-010-C). These two metastases were present at the time of resection of the primary tumor. Following treatment with FOLFIRI/bevacizumab the liver metastasis reduced, but the lung lesion remained stable. This patient was also treated with FOLFIRI/bevacizumab between the third (lung REACT-010-D) and fourth (liver REACT-010-E) metastases, but received no interval treatment between resection of the lung lesions (i.e. REACT-010-C and D).

Metastases in the liver share the same mutational profile for the driver mutations along with some private mutations in each (Fig. 4 and Supplementary Fig. 4). This suggests the possibility of dissemination of the founding clusters from the same region of the primary tumor sharing their ancestral sub-clonal structure. In contrast, for two lung metastatic tumors, which are only one-month apart, each harbor an exclusive driver mutation which is absent in the other. These are *APC* Q1349* (depicted by orange color in Fig. 4), which is present only in REACT-010-C, and a start loss mutation in *TP53* (depicted by dark blue in Fig. 4), which is present only in REACT-010-D. The most plausible scenario here is that the two metastases have originated from two clusters of cells disseminating from different regions of the primary tumor with different sub-clonal structures. The clinical course may explain why the same *APC* mutation is present in the liver resected samples and first lung lesion (REACT-010-C).

The similar profiles of driver mutations in the liver lesions (B and E) suggest that although these metastases manifested at different times, their founding cell-clusters disseminated around the same time. This pattern can be explained by dormancy of the disseminated cells that formed the second liver metastasis, or simply by differential growth rates of seeded cells, which formed the metastases. Our targeted deep-sequencing results confirm the reconstructed clonal structure (Supplementary Fig. 4). Figure 4 represents the possible scenario for the evolutionary trajectories within primary and metastatic lesions for patient REACT-010. The clustering schemes within these samples are represented in Supplementary Fig. 4.

The clustering and changes in the sub-clone population proportions for all the other patients with primary tumor are represented in Supplementary Fig. 3.

### Patients with no primary tumor available for analysis

Patient REACT-011 had two metastatic tumors in lung (4-months apart), with no available tissue from the primary tumor. Treatment with chemoradiation (5FU) + adjuvant FOLFOX was administered before development and resection of the first metastasis, however, no treatment was administered between metastases. Mutations in driver genes (nonsense mutations in both *APC* and *TP53*) are consistently present in both lesions. The tumors did not have mutations in any RAS pathway gene, but they share a missense mutation in the epigenetic modifier gene KMT2C, which shows high allele frequency in both lesions. Presence of the truncating *TP53* mutation may explain chemotherapy resistance. The shift in the allele frequencies of all the mutations towards higher values in the lung metastasis REACT-011-C suggests that relatively higher allele frequency of the main driver mutations in this tumor could be due to differences in tumor content between the two metastatic samples (40% vs 60%) (Supplementary Fig. 5A–C).

## Discussion

Our study is the first that applies a combination of whole-exome and ultra-deep targeted sequencing to the primary colorectal cancers as well as matched series of multiple metastatic lesions from individual patients. While our findings support previous studies highlighting the persistence of known clinically-relevant mutations between primary and metastatic CRC lesions^10,11^, we showed that, when present in the primary tumor, clinically-relevant *KRAS* and *NRAS* mutations are transmitted to the metastatic lesions of different organs and likely represent ubiquitous driver mutations^43^. Systemic therapies may apply selective pressure to the clonal composition of tumors, thereby contributing to resistance based upon changes in genomic aberrations. In EGFR-mutated non-small cell lung cancer (NSCLC), biopsies upon disease progression following targeted treatment have shown emergence of resistant clones in over 50% of cases, and altered histological subtype in 14%^44^. Treatment with temozolomide may influence the genomic tumor profile in patients with recurrent glioma, facilitating evolution to high-grade disease^45^. In CRC, analysis of cell lines and biopsy tissue from patients with tumors previously wild-type for *KRAS* mutations has shown that cetuximab treatment results in the emergence of *KRAS*-mutated sub-clones, as detected by analyzing circulating tumor DNA (ctDNA)^11^. However, our results did not reveal such emergence of RAS mutations in metastatic lesions, possibly due to small sample size. In NSCLC, higher *EGFR*-mutation allele frequency has been shown to correlate with response to EGFR-tyrosine kinase inhibitors^46,47^. In the event of development of appropriate targeted therapies for *KRAS*-mutated CRC, our results may have similar relevance. Notably, however, in the lung metastasis of patient REACT-008 we observed that cells affected by *mTOR* or *PIK3CA* mutations were eliminated following chemotherapy. Likewise, our mutational signature analysis indicated that interval chemotherapy can alter exonic mutational profiles of metastatic CRC tumors by promoting the enrichment or depletion of certain mutational signatures. These findings extend our knowledge about the effect of systemic therapy on the genomic architecture of metastatic tumors, beyond the status of *KRAS* and *NRAS* mutations, with potential translational implications. Furthermore, we also investigated mutational profiles of primary and an anastomotic recurrence tumor from a patient (patient 009) in our study. Interestingly, these tumors exhibited different mutational landscapes as WES revealed a hypermutated genome only in the primary and not in the recurrent tumor. Given that anastomotic recurrences are rare events as compared to metastasis in colorectal cancer patients, we found one recent study that compared mutations between primary CRC and anastomotic recurrences. By examining mutations of candidate genes, it has been suggested that anastomotic recurrences are clonally related to primary tumors^48^. However, this study included only microsatellite stable tumors, whereas tumor 9A in our study is a hypermutated tumor with mutations in *MSH2* and *MSH5* genes, suggesting that this tumor is affected by microsatellite instability. Further investigation may be required to understand the evolution of anastomotic recurrences where microsatellite stability differs.

Development of malignancy through clonal expansion may occur due to somatic mutations in key genes, for example in *KRAS* and *NRAS* in CRC, leading to dysregulation of the normal homeostatic mechanisms. Mutations conferring growth advantage are drivers of the carcinogenic and metastatic process and provide clinically-relevant targets for treatment^49^. Across tumor sites, genomes of disseminated cancer cells may have similarities at onset of metastatic disease. However, substantial changes in the genetic composition occur spatially and over time. It is therefore plausible that the expansion of aggressive driver clone(s), and emergence of relevant sub-clones, correlate with development of incurable disease and potential therapeutic resistance^50–53^. We were able to study these patterns in the mutational profile of patient REACT-10 for whom in addition to the primary tumor, four distinct metastatic specimens were available for mutational analysis. Our analysis revealed a similar mutational signature profile for the two liver metastases that was different from those observed in lung metastases. Furthermore, driver gene mutations were constantly present with high frequency in both liver lesions, a pattern which was not the same in lung metastatic tumors. Given that the liver metastases are at the two ends of the disease course in this patient, our results suggest that while two liver metastases were most likely originated from a common cell cluster in the primary tumor, the disseminated cells that formed the second liver metastasis experienced a dormant phase or a much slower growth rate before manifesting. The original seeding clusters may have resided in the same region within the primary tumor or may originate from different regions. The latter can be explained by ‘early cell mixing’ phenomenon, which has recently been shown for CRC^54^. These findings highlight the complex nature of evolution of metastatic colorectal cancers, and the importance of dormancy in this context. The mechanisms that underlie tumor dormancy and reactivation in metastatic CRC are not well known; however, a recent study suggests that non-genetic factors including chemotherapy play key roles here^55^.

Although presence of *KRAS* mutations using standardized clinical techniques may predict for resistance to anti-EGFR therapy, responses in *KRAS* wild-type tumors are not universal (single-agent response rates of 10–41%)^2,56,57^. Reasons for this are incompletely understood. Use of alternative technologies may detect low-level, or less frequent, *KRAS* mutations of uncertain clinical significance, undetectable by standard direct-sequencing and may also detect regional intratumor heterogeneity possibly directing treatment resistance^58–60^. Use of ultra-deep sequencing in our patient cohort ensured high sensitivity for *KRAS* and additional driver mutation analysis and permitted accurate quantification of mutant alleles in each specimen, necessary to study dynamics of mutational patterns throughout disease course. However, sampling was limited to a single region within each tumor specimen, precluding the evaluation of geographic heterogeneity within each lesion.

Data from our patient cohort highlights tumors lacking known clinically-relevant *KRAS* mutations with a complex, heterogeneous mutational landscape characterized by the presence of non-RAS mutations, as well as other *KRAS* mutations of uncertain clinical significance. We observed in tumors of three out of seven patients wild-type for *KRAS* codon 12 and 13, that *APC* was affected by protein-truncating mutations, an early event in CRC tumorigenesis^61^. Additional research including larger number of samples is warranted to investigate the mutational landscape of tumors that lack *KRAS* codon 12 and 13 mutations.

Sub-classification of CRC has been challenging and, despite the identification of a number of prognostic subgroups^62,63^, understanding of key driver mutations remains critical for optimal treatment of metastatic CRC. As there is greater understanding of the molecular landscape of CRC and as non-invasive methods of molecular testing become more frequently ultilised^64,65^ targeted ultra-deep sequencing of driver mutations provides a powerful tool to monitor tumor behavior in clinic, and to identify recurrent lesions, molecularly distinct from primary tumors. This technique demonstrates high sensitivity for mutation detection and its use in this study has confirmed the persistence of clinically-relevant mutations between primary tumor and metastatic lesions. Additionally, this work has highlighted the impact of latency and systemic treatment pressure effects in the evolution of metastatic CRC lesions, resulting in modulation of mutation frequency for driver mutations. By enhancing our knowledge about the behavior of driver mutations, these findings may have translational value for future clinical management of patients.

## Supplementary information

Supplementary Figures.

Supplementary Dataset 1.

Supplementary Dataset 2.

Supplementary Dataset 3.

Supplementary Dataset 4.

Supplementary Dataset 5.
