# Supplementary material for: Latency and interval therapy affect the evolution in metastatic colorectal cancer
Source: Sci Rep. 2020 Jan 17;10:581. doi: 10.1038/s41598-020-57476-y (PMC6969060; doi:10.1038/s41598-020-57476-y)
Supplement: Supplementary file 1 — Supplementary Figures. [file 41598_2020_57476_MOESM1_ESM.pdf]

Latency and interval therapy affect the evolution in metastatic colorectal cancer

Hamid Nikbakht[1,2], Selin Jessa[1,2], Mahadeo A. Sukhai[3], Madeleine Arseneault[1,2], Tong Zhang[3], Louis Letourneau[2], Mariam Thomas[3], Mathieu Bourgey[2], Michael. H. A. Roehrl[4,5], Robert Eveleigh [2], Eric X. Chen[3], Monica Krzyzanowska[3], Malcolm J. Moore[3], Amanda Giesler[3], Celeste Yu[3], Philippe L. Bedard[3], Suzanne Kamel-Reid[3], Jacek Majewski[1,2], Lilian L. Siu[3], Yasser Riazalhosseini[1,2], Donna M. Graham[3]

1. Department of Human Genetics, McGill University, Montreal, Québec, Canada
2. McGill University and Génome Québec Innovation Centre, Montreal, Québec, Canada
3. Princess Margaret Cancer Centre, Toronto, Ontario, Canada
4. UHN Program in BioSpecimen Sciences, Toronto General Hospital, Toronto, Ontario, Canada
5. Department of Pathology, Toronto General Hospital, Toronto, Ontario, Canada

Email addresses:

h.nikbakht@gmail.com, selin.jessa@mail.mcgill.ca, m.sukhai@utoronto.ca, madeleine.arseneault@mcgill.ca, tong.zhang@uhn.ca, lletourn49@gmail.com, mariam.thomas@alum.utoronto.ca, mathieu.bourgey@mcgill.ca, roehrlm@mskcc.org, eveleigh.rjm@gmail.com, Eric.Chen@uhn.ca, monika.krzyzanowska@uhn.ca, malcolm.moore@bccancer.bc.ca, amanda.giesler@uhn.ca, celeste.yu@uhn.ca, Philippe.Bedard@uhn.ca, suzanne.kamel-reid@uhn.ca, jacek.majewski@mcgill.ca, Lillian.Siu@uhn.ca, yasser.riazalhosseini@mcgill.ca, drdonnagraham@gmail.com

Corresponding Author:

Yasser Riazalhosseini

Department of Human Genetics,

McGill University, Montreal, Québec, Canada

Email: Yasser.riazalhosseini@mcgill.ca

Supplementary Figure 1. CONSORT diagram showing patient selection.

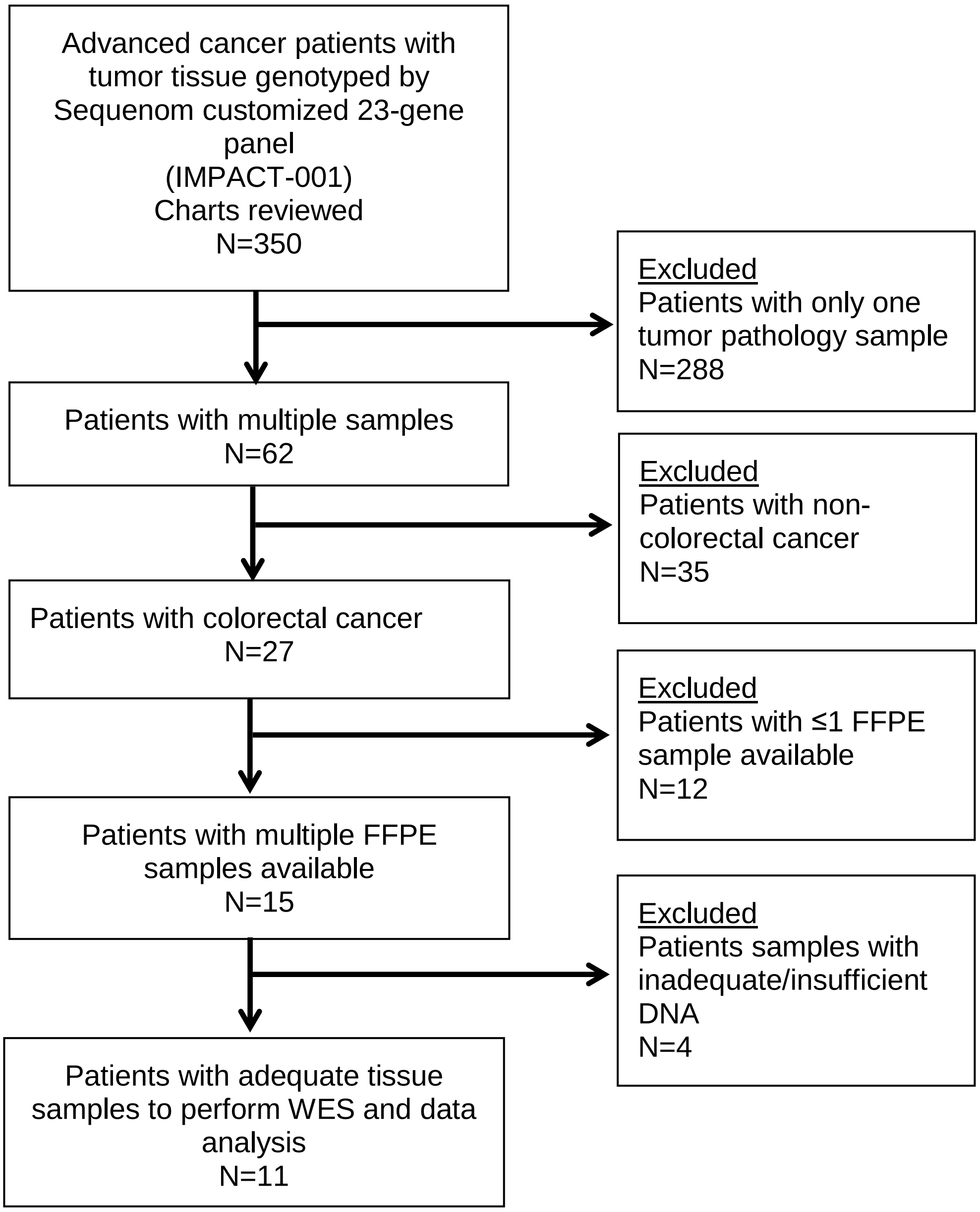

FFPE = formalin-fixed paraffin-embedded; WES = Whole exome sequencing

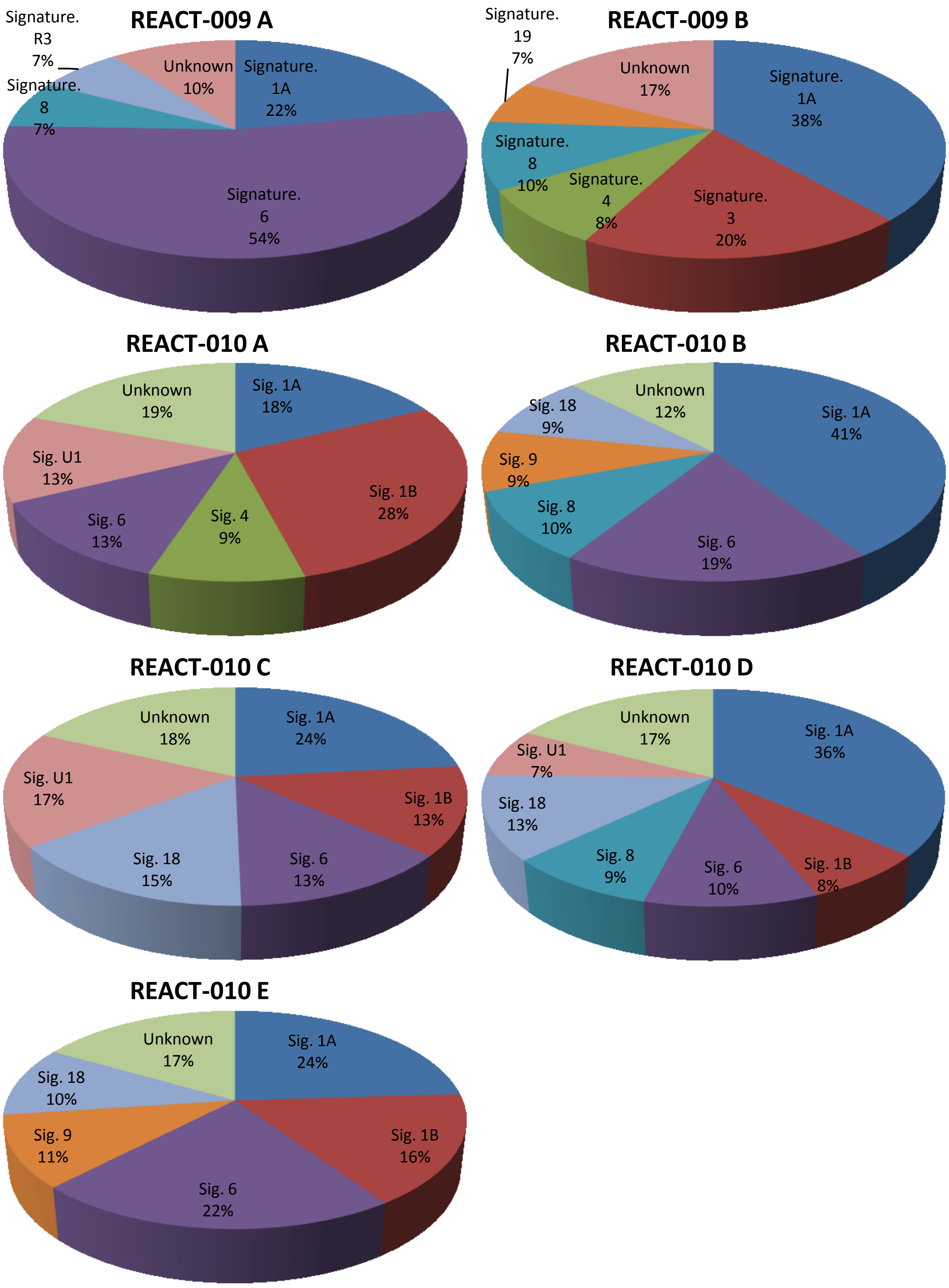

Supplementary Figure 2) Mutation signature in two patients REACT009 and REACT010 and their metastatic lesions

A)

B

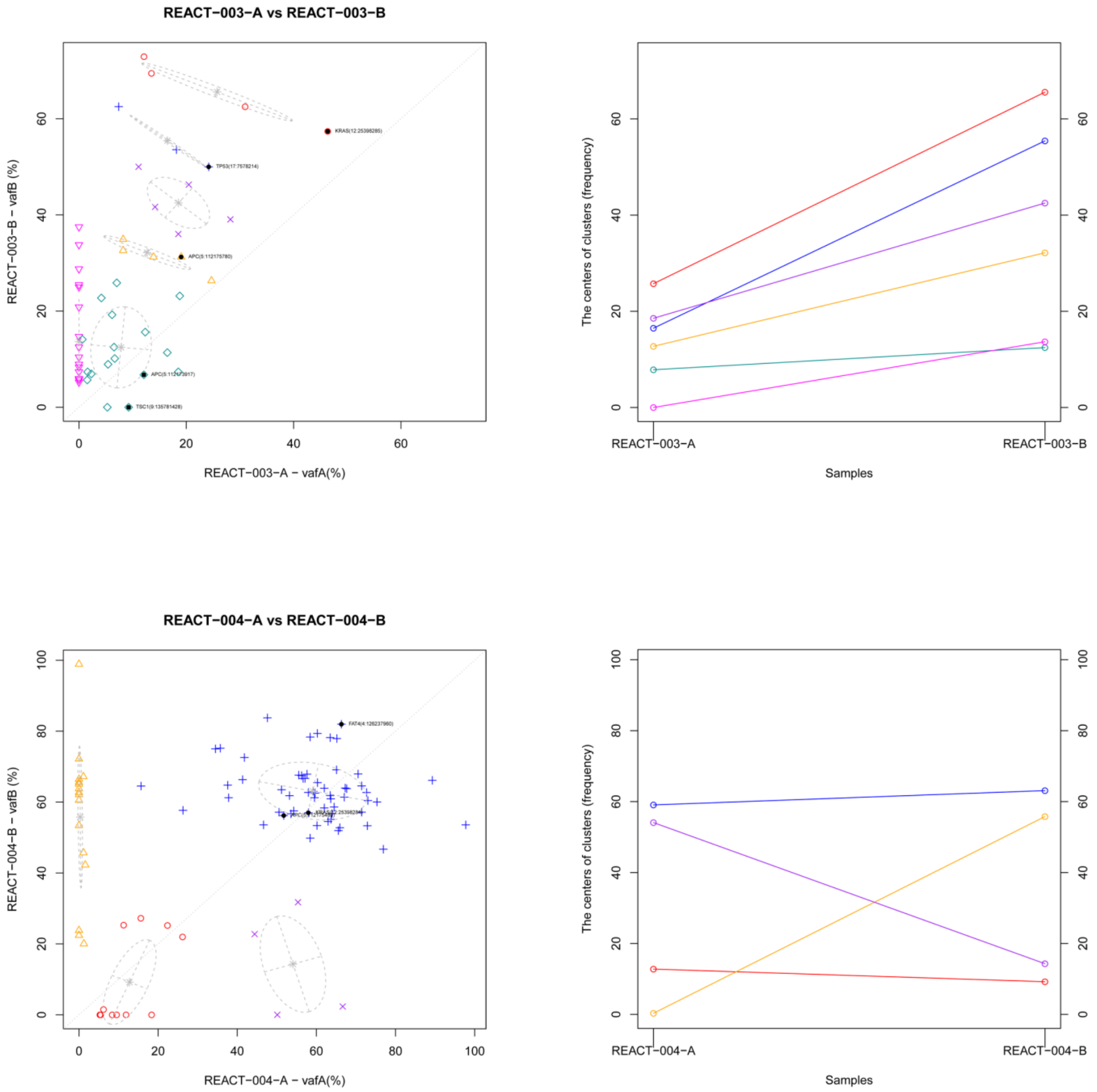

REACT−003−A vs REACT−003−B
REACT−003−B − vafB (%)
REACT−003−A − vafA(%)
KRAS(12:25398285)
TP53(17:7578214)
APC(5:112175780)
TSC1(9:135781428)
The centers of clusters (frequency)
REACT−003−A
REACT−003−B
Samples
REACT−004−A vs REACT−004−B
REACT−004−B − vafB (%)
REACT−004−A − vafA(%)
The centers of clusters (frequency)
REACT−004−A
REACT−004−B
Samples

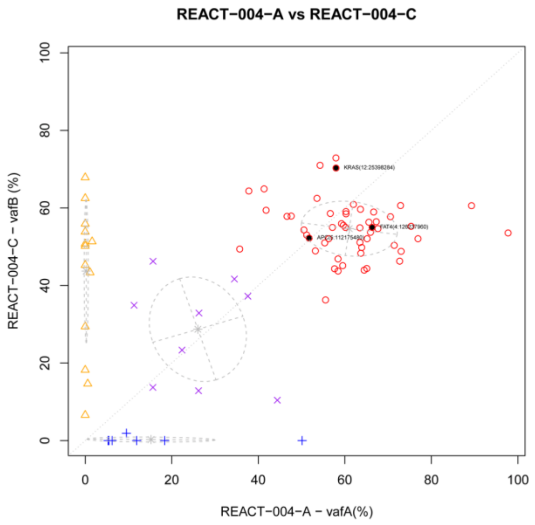
REACT−004−A vs REACT−004−C
REACT−004−C − vafB (%)
REACT−004−A − vafA(%)

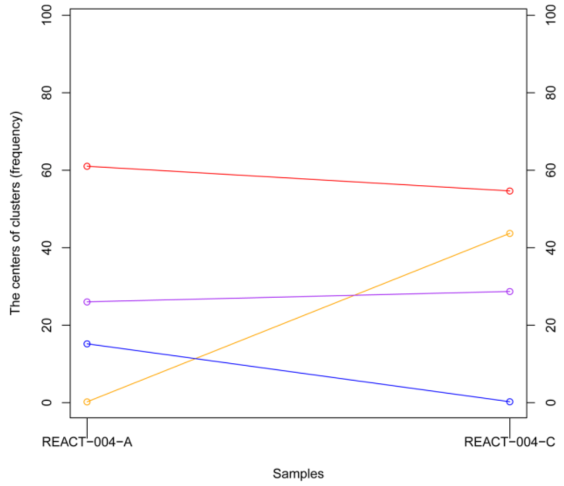
The centers of clusters (frequency)
REACT−004−A
REACT−004−C
Samples

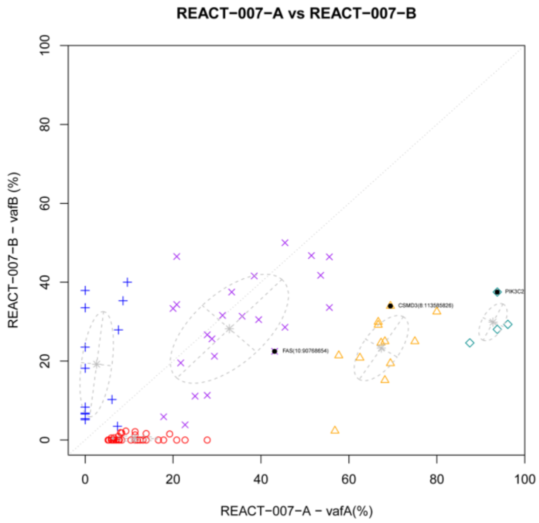
REACT−007−A vs REACT−007−B
REACT−007−B − vafB (%)
REACT−007−A − vafA(%)

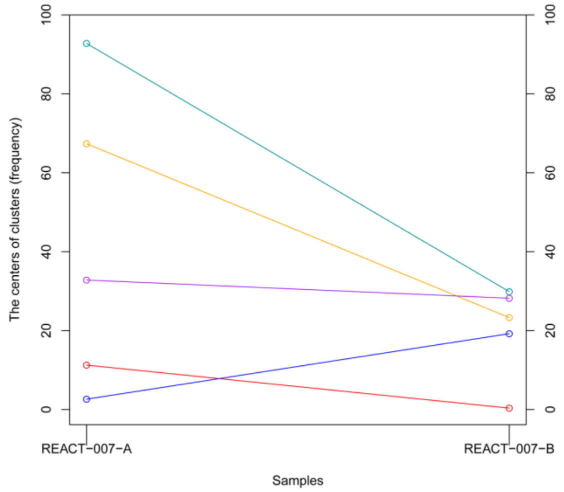
The centers of clusters (frequency)
REACT−007−A
REACT−007−B
Samples

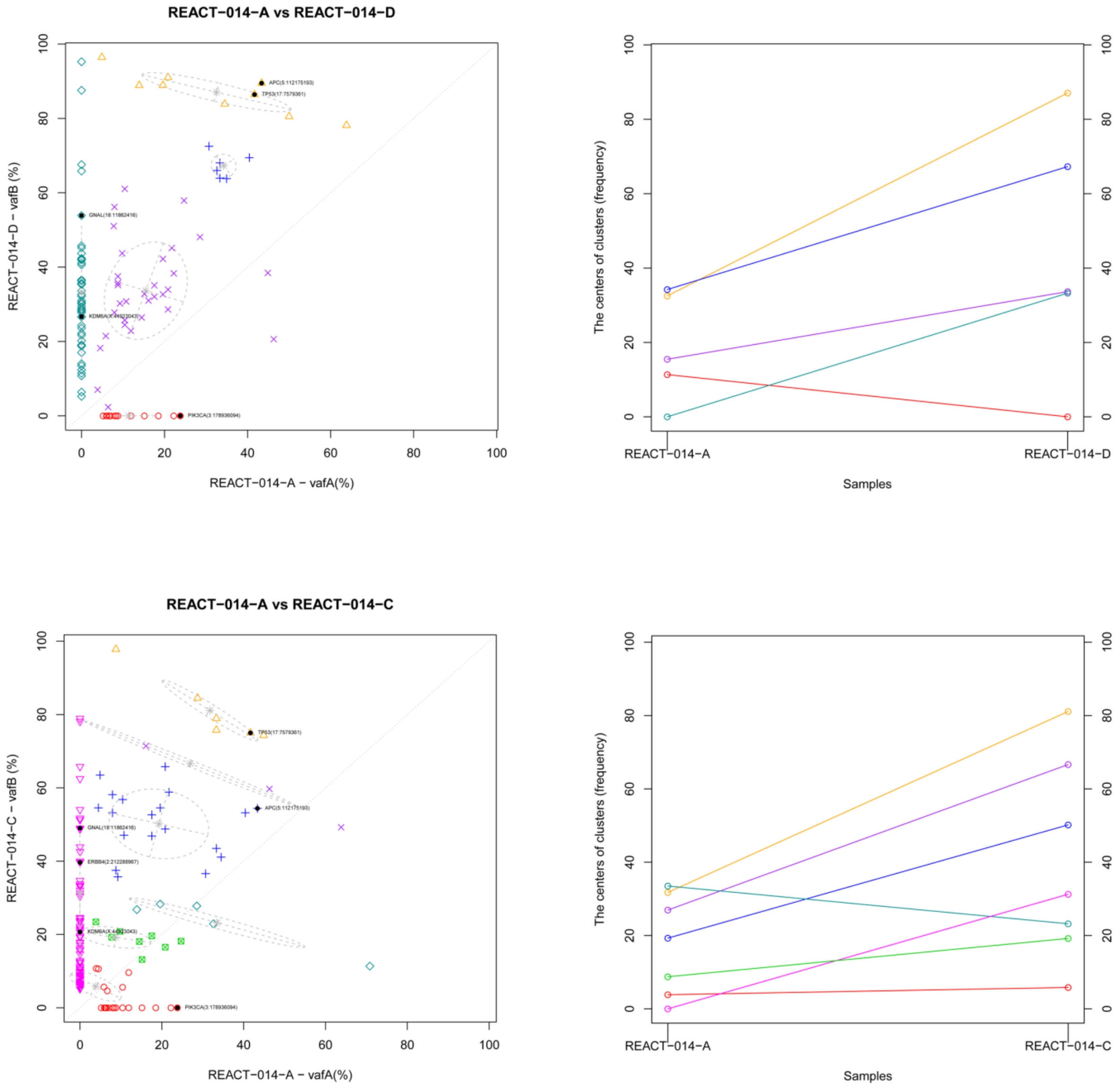

Supplementary Figure 3) A) Clustering of the mutations between the primary and different metastatic lesions in other patients with primary tumor B) Changes in the cell population proportions in different clusters between primary and different metastasis in these patient

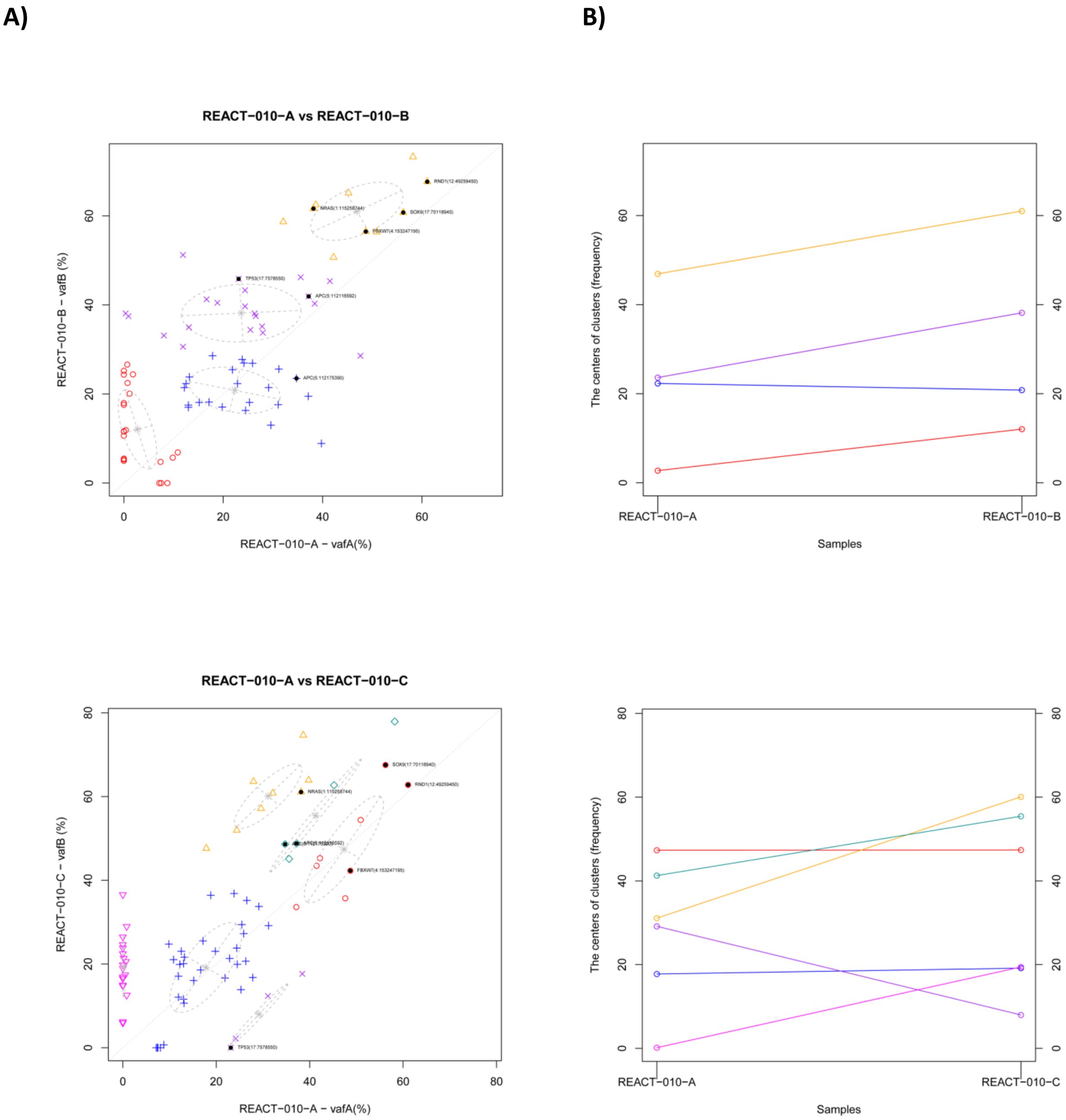
A)
B)
REACT−010−A vs REACT−010−B
REACT−010−B − vafB (%)
REACT−010−A − vafA(%)
RND1(12:49259450)
NRAS(1:115258744)
SOX9(17:70118940)
FBXW7(4:153247195)
TP53(17:7578550)
APC(5:112116592)
APC(5:112175390)
The centers of clusters (frequency)
Samples
REACT−010−A
REACT−010−B
REACT−010−A vs REACT−010−C
REACT−010−C − vafB (%)
REACT−010−A − vafA(%)
SOX9(17:70118940)
RND1(12:49259450)
NRAS(1:115258744)
FBXW7(4:153247195)
TP53(17:7578550)
The centers of clusters (frequency)
Samples
REACT−010−A
REACT−010−C

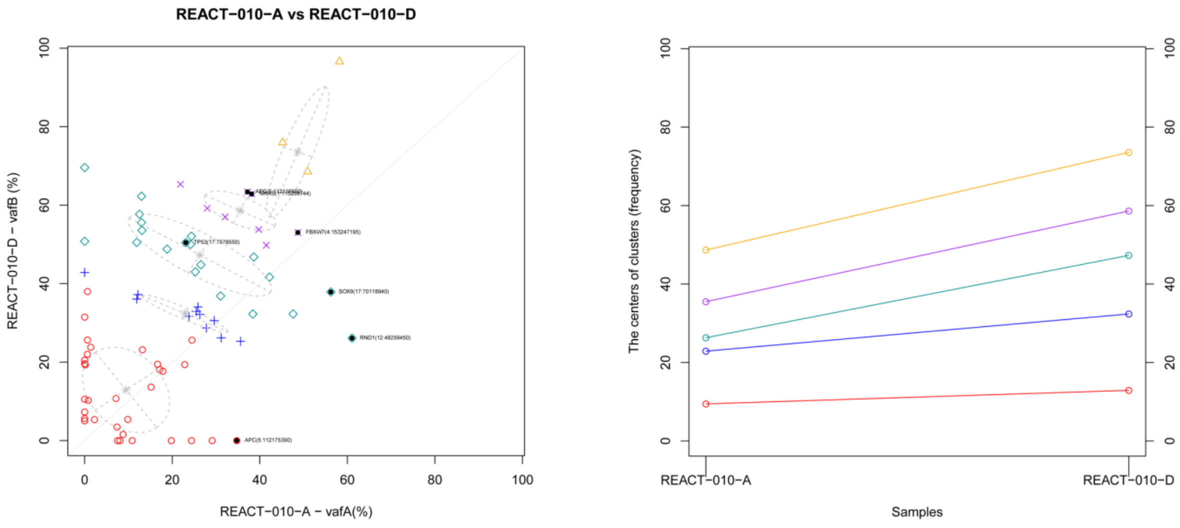
REACT−010−A vs REACT−010−D
REACT−010−D − vafB (%)
REACT−010−A − vafA(%)
TP53(17:7578550)
FBXW7(4:153247195)
SOX9(17:70118940)
RND1(12:49259450)
APC(5:112175390)
The centers of clusters (frequency)
REACT−010−A
REACT−010−D
Samples

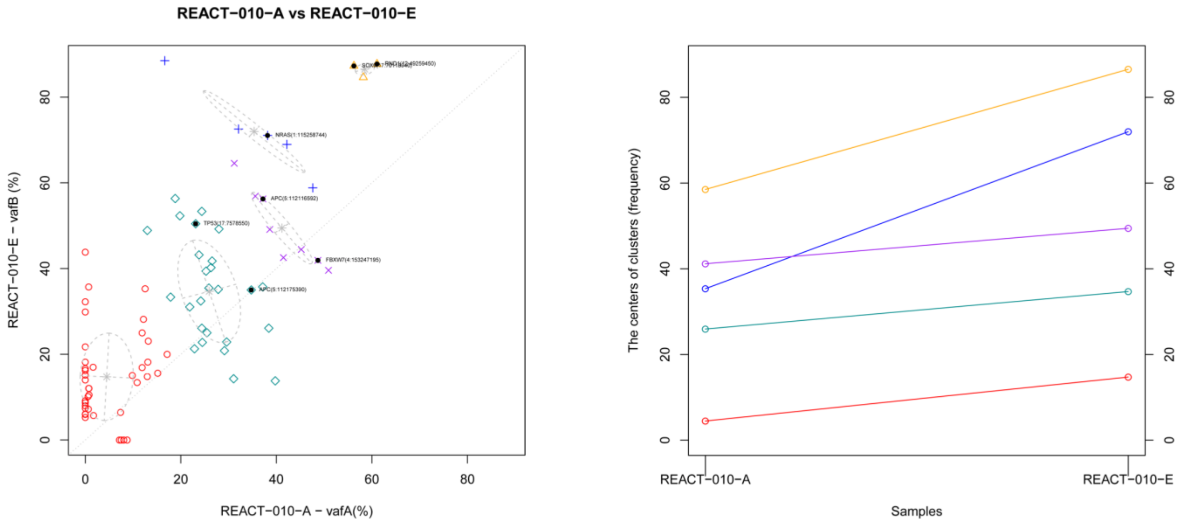
REACT−010−A vs REACT−010−E
REACT−010−E − vafB (%)
REACT−010−A − vafA(%)
NRAS(1:115258744)
APC(5:112116592)
TP53(17:7578550)
FBXW7(4:153247195)
APC(5:112175390)
The centers of clusters (frequency)
REACT−010−A
REACT−010−E
Samples

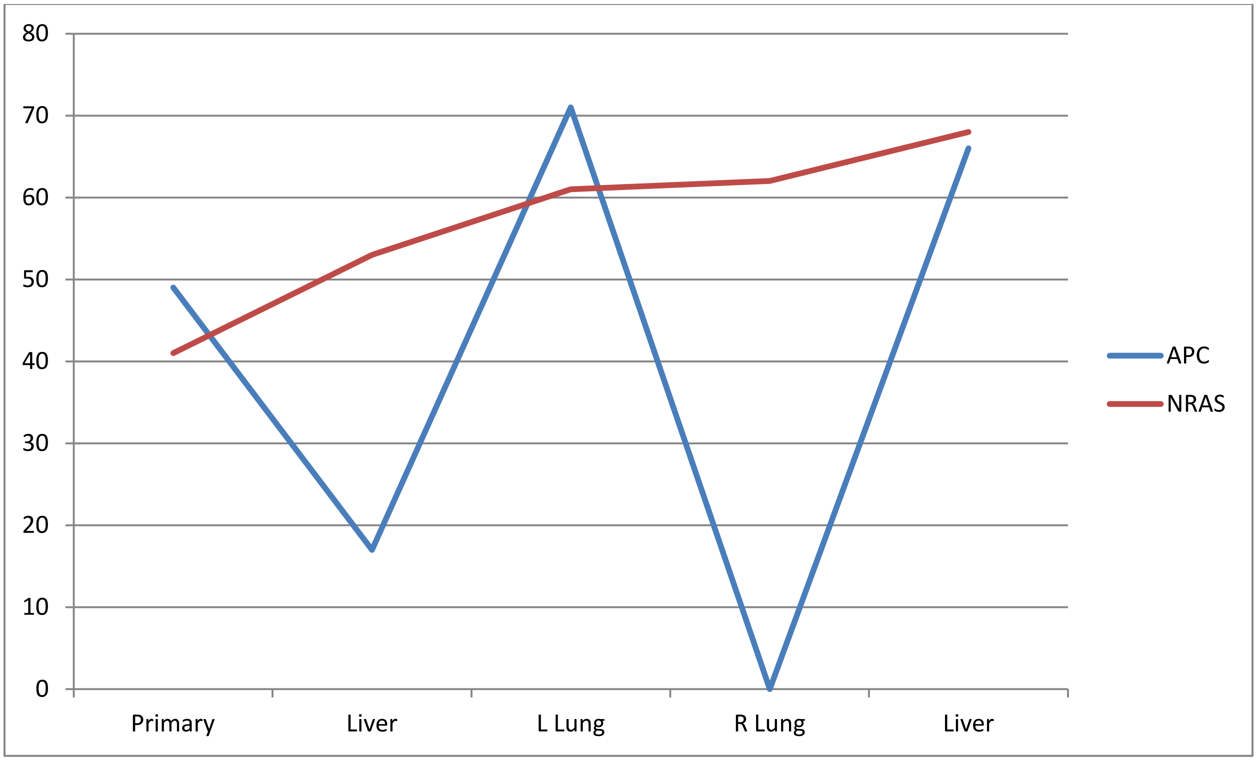

Supplementary Figure 4) A) Clustering of the mutations between the primary and different metastatic lesions in patient REACT-010 B) Changes in the cell population proportions in different clusters between primary and different metastasis in this patient. C) Deep sequencing results shows an enrichment of NRAS codon 13 mutated cells emerging in metastatic disease following administration of interval systemic chemotherapy. Interestingly between left Lung and right Lung that there were no chemotherapy administration, the rate of the increase has slowed down.

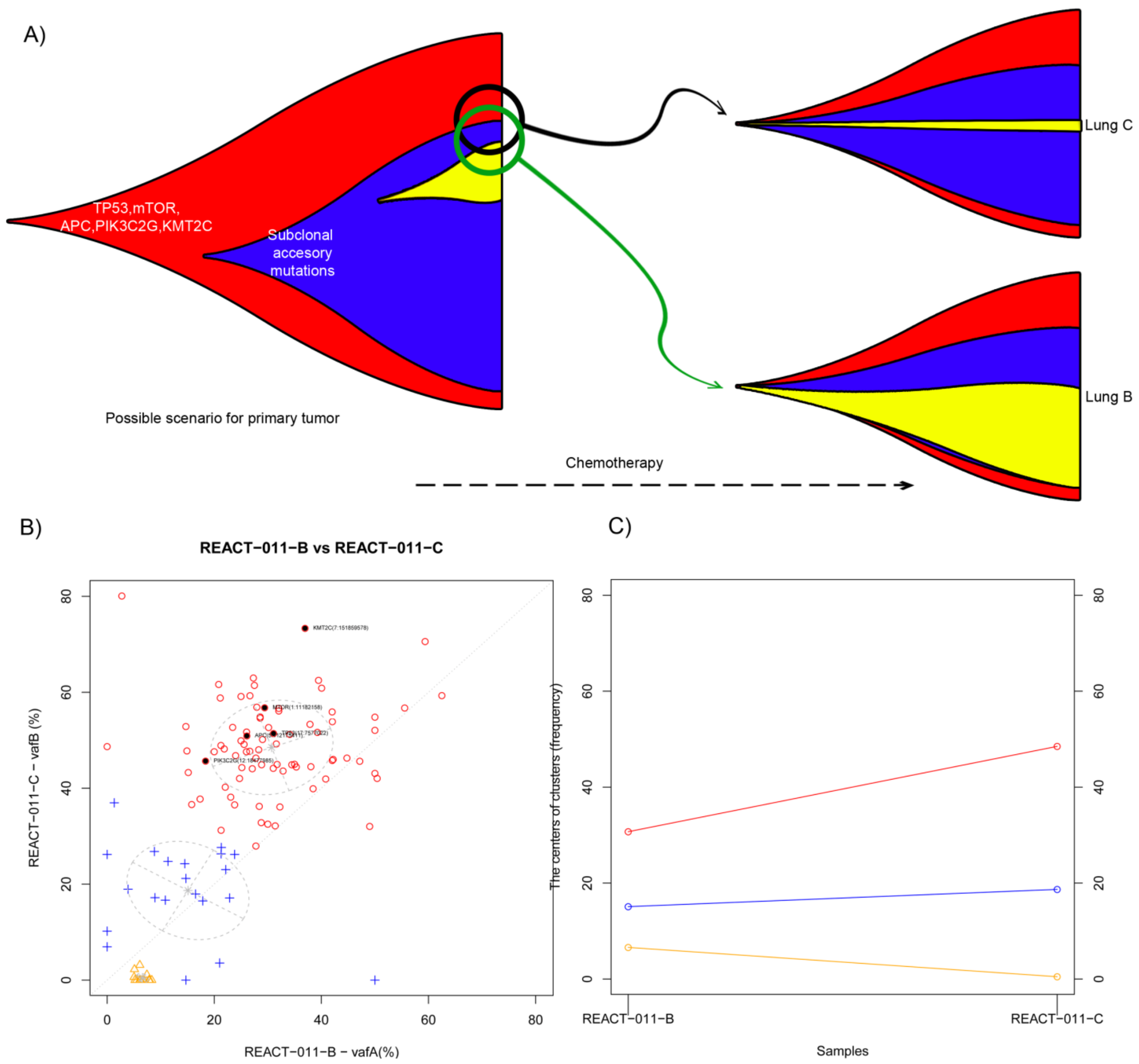

Supplementary Figure 5) A) Proposed scenario for the evolution within the primary and metastatic lesion in patient REACT-011 B) Clustering of the mutations between the primary and metastatic lesions C) Changes in the cell population proportions in different clusters between primary and metastasis
